# Neuroprotective Effect of Dexmedetomidine on Hyperoxia-Induced Toxicity in the Neonatal Rat Brain

**DOI:** 10.1155/2015/530371

**Published:** 2015-01-13

**Authors:** Marco Sifringer, Clarissa von Haefen, Maria Krain, Nadine Paeschke, Ivo Bendix, Christoph Bührer, Claudia D. Spies, Stefanie Endesfelder

**Affiliations:** ^1^Department of Anesthesiology and Intensive Care Medicine, Charité-Universitätsmedizin Berlin, Campus Virchow-Klinikum, 13353 Berlin, Germany; ^2^Department of Pediatrics I, Neonatology, University Hospital Essen, 45122 Essen, Germany; ^3^Department of Neonatology, Charité-Universitätsmedizin Berlin, 13353 Berlin, Germany

## Abstract

Dexmedetomidine is a highly selective agonist of *α*2-receptors with sedative, anxiolytic, analgesic, and anesthetic properties. Neuroprotective effects of dexmedetomidine have been reported in various brain injury models. In the present study, we investigated the effects of dexmedetomidine on neurodegeneration, oxidative stress markers, and inflammation following the induction of hyperoxia in neonatal rats. Six-day-old Wistar rats received different concentrations of dexmedetomidine (1, 5, or 10 *µ*g/kg bodyweight) and were exposed to 80% oxygen for 24 h. Sex-matched littermates kept in room air and injected with normal saline or dexmedetomidine served as controls. Dexmedetomidine pretreatment significantly reduced hyperoxia-induced neurodegeneration in different brain regions of the neonatal rat. In addition, dexmedetomidine restored the reduced/oxidized glutathione ratio and attenuated the levels of malondialdehyde, a marker of lipid peroxidation, after exposure to high oxygen concentration. Moreover, administration of dexmedetomidine induced downregulation of IL-1*β* on mRNA and protein level in the developing rat brain. Dexmedetomidine provides protections against toxic oxygen induced neonatal brain injury which is likely associated with oxidative stress signaling and inflammatory cytokines. Our results suggest that dexmedetomidine may have a therapeutic potential since oxygen administration to neonates is sometimes inevitable.

## 1. Introduction

Premature birth is the leading cause of child mortality and morbidity in preterm infants because most organs and also the antioxidant enzyme system are not fully developed structurally and functionally [1, 2]. Despite advances in perinatal medicine, whereby the chances of survival of premature infants could be significantly increased, in preterm infants with low birth weight impairment of brain development is often observed [3, 4].

A general anesthesia is sometimes essential for premature infants with medical indication. In pediatric anesthesia usually the same anesthetics and adjuvants are used as in adults. NMDA antagonists like ketamine and/or GABA_A_ receptor agonists, such as benzodiazepines, barbiturates, isoflurane, or propofol, are employed [5, 6]. In particular, premature and newborn infants demonstrate a significantly higher risk of anesthesia [7] and perioperative morbidity and mortality is increased due to the immature organ systems [8]. In addition to required medical interventions under sedation also high oxygen concentrations are a major problem. In recent years, experimental studies and clinical observations showed that oxygen, which is widely used in neonatal intensive care for treatment of respiratory distress, triggers a disruption of intracellular redox homeostasis and disturbed neurological development of preterm infants [9–11]. As we demonstrated recently in experimental models, this disturbance can induce oxidative stress by modulation of the glutathione ratio and an increasing lipid peroxidation [12–15], inflammation by increased levels of proinflammatory cytokines [13, 15, 16], leading to increased neurodegeneration [12, 16, 17], and inhibition of neuronal maturation [17, 18] in the developing brain.

Dexmedetomidine is a potent and highly selective agonist of *α*2-receptors with sedative, anxiolytic, analgesic, and anesthetic properties [19–21]. In addition, it is generally reported that dexmedetomidine has neuroprotective effects in different animal models [22–27] and minimal side effects on the respiratory tract and the gastrointestinal function that minimizes the exposure to other narcotics and benzodiazepines [23, 28–30]. Recent studies suggest the *α*2-adrenoceptor agonist dexmedetomidine to attenuate anesthetic agent induced neuroapoptosis [26, 31, 32] and it appears for long term sedation as an alternative to midazolam and propofol [33, 34]. Advantages of dexmedetomidine are shorter ventilation and recovery times and lower hypertension and tachycardia. In particular, the shortening of the duration of ventilation seems to be relevant in connection with the higher oxygen toxicity by prolonged ventilation and the associated development of chronic lung disease and the consequent motor and cognitive deficits in preterm born infants. First results of clinical trials in preterm infants revealed a decrease in the duration of mechanical ventilation by half and no need for additional sedative administration [35, 36].

The aim of this study was to investigate the effect of different concentrations of dexmedetomidine to the immature brain in a neonatal rat model of oxygen toxicity on neurodegeneration, oxidative stress markers, and the expression of the proinflammatory cytokine IL-1*β*.

## 2. Materials and Methods

### 2.1. Animals and Drug Administration

All procedures were approved by the State Animal Welfare Authorities (LAGeSo G0145/13) and followed institutional guidelines. Six-day-old Wistar rats from time-pregnant mothers were obtained from Charité-Universitätsmedizin Berlin (Germany) and randomly assigned to cages and treatment.

Dexmedetomidine (DEX; dexdor, Orion Pharma, Espoo, Finland) was dissolved in phosphate buffered saline. Three doses of the drug (1, 5, and 10 *μ*g/kg body weight) were used and all injections were given intraperitoneally (i.p.) as a fixed proportion of body weight (100 *μ*L/10 g). The rat pups are divided into different biological groups (description with the relevant experimental abbreviations): (1) control group (CON; 21% O_2_, room air) with 0,9% saline, (2) verum group (21% O_2_) with 1 *μ*g/kg DEX (DEX1), (3) verum group with 5 *μ*g/kg DEX (DEX5), (4) verum group with 10 *μ*g/kg DEX (DEX10), (5) hyperoxia group (HY; 80% O_2_, OxyCycler BioSpherix, Lacona, NY) with 0,9% saline, (6) hyperoxia with 1 *μ*g/kg DEX (HYDEX1), (7) hyperoxia with 5 *μ*g/kg DEX (HYDEX5), and (8) hyperoxia with 10 *μ*g/kg DEX (HYDEX10), each with a number of six animals per group and different gender. For hyperoxia or normoxia exposure, pups were kept together with their dams. Saline or DEX was administrated once 15 min before the start of oxygen exposure.

### 2.2. Tissue Preparation

At 24 h of exposure the animals were anaesthetized with an i.p. injection of ketamine (50 mg/kg), xylazine (10 mg/kg), and acepromazine (2 mg/kg) 5 min before being perfused. For molecular analysis, pups were transcardially perfused with normal saline (pH 7.4) and then decapitated, the olfactory bulb and cerebellum were removed, and brain hemispheres were snap-frozen in liquid nitrogen and stored at −80°C. For immunohistochemical analysis, animals were perfused with PBS followed by perfusion with 4% paraformaldehyde at pH 7.4 and the brains were postfixed at 4°C for 3 days, embedded in paraffin, and processed for histological staining.

### 2.3. DNA Fragmentation Assay

Paraffin-embedded sections were cut (5 *μ*m), deparaffinized in Roti-Histol (Carl Roth, Karlsruhe, Germany) twice for 10 min each, rehydrated in descending ethanol series, and rinsed in phosphate buffered saline for 3 min each at room temperature. After deparaffinization of sections an* in situ* detection of cells with DNA-strand breaks was performed by the TUNEL labeling method using a TdT-FragEL DNA fragmentation detection kit (Millipore, Darmstadt, Germany) according to the manufacturer's instructions. Negative controls were performed by substituting Tris-buffered saline for the TdT enzyme.

The TUNEL positive cells were analyzed in frontal cortex (FC), retrosplenial cortex (RSC), hypothalamus (HTH), thalamus (TH), and the hippocampus. Sections were viewed by light microscopy while blinded using a LEICA DM 2000 microscope equipped with a 200x magnification. TUNEL positive cells were counted in the anatomical regions of the brain in up to twelve different sections per animal and region.

### 2.4. Determination of Total Glutathione (GSH and GSSG)

Total glutathione (GSH and GSSG) was measured in brain homogenates using the thiol reagent 5,5′-dithiobis-2-nitrobenzoic acid (DTNB) as shown previously [14]. In brief, for the determination of reduced glutathione (GSH) and oxidized glutathione (GSSG), the brains were homogenized and the homogenates were treated with a mixture of metaphosphoric acid, EDTA, and NaCl. After centrifugation, aliquots were taken for neutralization with disodium hydrogen phosphate followed by addition of DTNB. GSH was determined after reaction with DTNB in a spectrophotometer at 412 nm. For the determination of GSSG 4-vinylpyridine was added and then incubated for 1 hour at room temperature. 4-Vinylpyridine is able to mask the GSH content without interfering with the spectrophotometrical determination of GSSG at 412 nm. GSH and GSSG levels are reported as nmol/mg protein.

### 2.5. Measurement of Lipid Peroxidation

Lipid peroxidation was determined by the reaction of thiobarbituric acid with malondialdehyde (MDA), a product of lipid breakdown caused by oxidative stress as previously described [14]. A SUPELCOSIL LC-18-DB HPLC reversed-phase column (Sigma-Aldrich, Munich, Germany; 5 *μ*m particle size, 250 × 10 mm I.D.) was utilized for the detection of MDA levels in brain homogenates. The MDA level was determined by fluorescence (525/550 nm) with a 50 mM potassium phosphate buffer (pH 6.8) and 40% methanol mobile phase at 1.5 mL/min flow rate.

### 2.6. RNA Extraction and Semiquantitative Real-Time PCR

Total RNA was isolated from snap-frozen tissue by acidic phenol/chloroform extraction (peqGOLD RNAPure; PEQLAB Biotechnologie, Erlangen, Germany) and 2 *μ*g of RNA was reverse-transcribed. The PCR products of* IL*-*1β* and* hypoxanthine-guanine phosphoribosyltransferase* (*HPRT*, as internal standard) were quantified in real time, using dye-labeled fluorogenic reporter oligonucleotide probes and primers (Metabion, Munich, Germany) with the following sequences and corresponding GenBank accession numbers:* IL*-*1β* (NM_031512) sense 5′-AACAAAAATGCCTCGTGCTGTCT-3′, antisense 5′-TGTTGGCTTATGTTGTGTCCATTG-3′, probe 5′-ACCCATGTGAGCTGAAAGCTCTCC-3′;* HPRT* (NM_012583) sense 5′-GGAAAGAACGTCTTGATTGTTGAA-3′, antisense 5′-CCAACACTTCGAGAGGTCCTTTT-3′, and probe 5′-CTTTCCTTGGTCAAGCAGTACAGCCCC-3′. All probes were labeled at their 5′ ends with the reporter dye 6-carboxy-fluoresceine (FAM) and at their 3′ ends with the quencher dye 6-carboxy-tetramethylrhodamine (TAMRA). Real-time PCR and detection were performed in triplicate and repeated 3 times for each sample using a total reactive volume of 13 *μ*L which contained 6,5 *μ*L of 2x TaqMan Universal PCR Master Mix (Applied Biosystems, Foster City, CA, USA), 2.5 *μ*L of 1.25 *μ*M oligonucleotide mix, 0,5 *μ*L (0,5 *μ*M) of probe, and 50 ng of cDNA template. The PCR amplification was performed in 96-well optical reaction plates for 40 cycles with each cycle at 94°C for 15 s and 60°C for 1 min. The expression of* IL*-*1β* and* HPRT* was analyzed with the real-time PCR ABI Prism 7500 sequence detection system (Applied Biosystems) according to the 2^−ΔΔCT^ method [37].

### 2.7. Immunoblotting

Snap-frozen brain tissue was homogenized in RIPA buffer solution for protein extraction. The homogenate was centrifuged at 1,050 g (4°C) for 10 min, and the microsomal fraction was subsequently centrifuged at 17,000 g (4°C) for 20 min. After collecting the supernatant, protein concentrations were determined using the Pierce BCA kit (Pierce, Rockford, IL, USA) with a 30 min incubation at 37°C prior to spectrophotometry at 562 nm. Protein extracts (30 *μ*g per sample) were denatured in Laemmli sample loading buffer at 95°C, separated by 15% of sodium dodecyl sulfate polyacrylamide gel electrophoresis, and electrotransferred in transfer buffer to a nitrocellulose membrane (0.2 *μ*m pore, Protran; Schleicher & Schüll, Dassel, Germany). Nonspecific protein binding was prevented by treating the membrane with 5% nonfat dry milk in Tris-buffered saline/0.1% Tween 20 for 1 h at room temperature. Equal loading and transfer of proteins was confirmed by staining the membranes with Ponceau S solution (Fluka, Buchs, Switzerland). The membranes were incubated overnight at 4°C with rabbit polyclonal anti-IL-1*β* (17 kDa; 1 : 1000; PromoKine, Heidelberg, Germany). Horseradish peroxidase-conjugated secondary anti-rabbit antibody was diluted 1 : 2000 (Amersham Biosciences, Bucks, United Kingdom). Positive signals were visualized using the SuperSignal West Pico kit (Pierce) according to the manufacturer's directions and quantified using a ChemiDoc XRS+ system and the software Image Lab (Bio-Rad, Munich, Germany). Membranes were stripped and then washed, blocked, and reprobed overnight at 4°C with mouse anti-*β*-actin monoclonal antibody (42 kDa; 1 : 10.000; Sigma-Aldrich). Each experiment was repeated three times.

### 2.8. Statistical Analyses

All data are expressed as mean ± standard error of the mean (SEM). Groups were compared using a one-way analysis of variance (ANOVA), and significance was determined using Bonferroni's correction for multiple comparisons with independent sample *t*-test. A two-sided *P* value < 0.05 was considered to be significant. All graphics and statistical analyses were performed using the GraphPad Prism 6.0 software (GraphPad Software, La Jolla, CA, USA).

## 3. Results

### 3.1. Dexmedetomidine Ameliorates Hyperoxia-Induced Neurodegeneration in the Infant Brain

That oxidative stress is a trigger of cell death is well known. We have investigated the changes in oxidative stress on the apoptotic cell death by TUNEL assay. These investigations were carried out in the frontal cortex (FC) and retrosplenial cortex (RSC), in the deep gray matter (hypothalamus (HTH), thalamus (TH)), and in the hippocampus.

**Figure 1:**
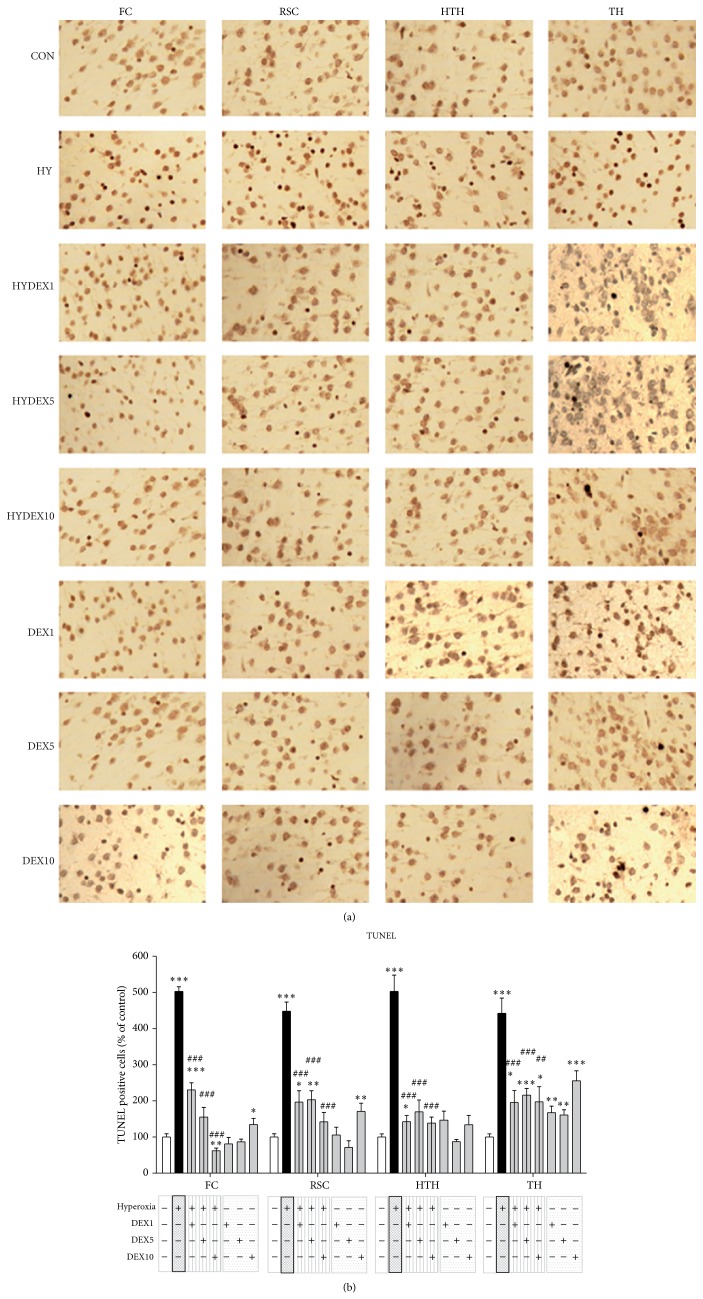
Apoptosis caused by hyperoxia is prevented by dexmedetomidine. (a) Representative TUNEL staining images (original magnification ×400) of rat brain frontal cortices (FC), retrosplenial cortices (RSC), hypothalamus (HTH), and thalamus (TH) of P7 control pups in room air without (CON) and with dexmedetomidine administration (DEX1, DEX5, and DEX10, corresponding to the concentrations of 1, 5, and 10 *μ*g/kg) and after 24 h of hyperoxia from P6 to P7 without (HY) and with dexmedetomidine administration (HYDEX1, HYDEX5, and HYDEX10). (b) Quantitation of TUNEL positive cells in the rat brain frontal cortices (FC), retrosplenial cortices (RSC), hypothalamus (HTH), and thalamus (TH) showed that relative to the control (white bars) hyperoxia at 24 h significantly increased these cell counts in cortex and deep grey matter (black bars). These levels were significantly decreased through systemic dexmedetomidine pretreatment (hatched grey bars; DEX 1, 5, and 10 *μ*g/kg). However, dexmedetomidine administration resulted in increased TUNEL positive cells in control rats most profound in TH (grey bars; DEX 1, 5, and 10 *μ*g/kg). Data are expressed relative to the normoxia-exposed control group (white bars, 100%). Bars represent mean + SEM; *n* = 6 per group; ^*^ *P* < 0.05, ^**^ *P* < 0.01, and ^***^ *P* < 0.001 versus normoxia/control; ^##^ *P* < 0.01 and ^###^ *P* < 0.001 versus hyperoxia.

In detail, exposure to 24 h of hyperoxia (HY) from P6 to P7 resulted in a large increase of TUNEL positive cells and the antiapoptotic ability of dexmedetomidine was able to be detected by a significant decrease in the investigated different concentrations (FC: HY 502.9 + 13.0% versus HYDEX1 230.7 + 19.5%, HYDEX5 155.0 + 27.2%, and HYDEX10 62.1 + 7.2%; RSC: HY 448.3 + 25.3% versus HYDEX1 196.8 + 31.2%, HYDEX5 203.4 + 24.6%, and HYDEX10 142.2 + 25.9%; HTH: HY 502.9 + 45.0% versus HYDEX1 142.6 + 16.9%, HYDEX5 169.9 + 32.5%, and HYDEX10 138.7 + 16.2%; TH: HY 442.4 + 42.3% versus HYDEX1 195.5 + 33.2%, HYDEX5 215.6 + 18.7%, and HYDEX10 197.5 + 41.9%) compared to control animals (CON; Figures 1(a) and 1(b)). Particularly DEX10 under hyperoxic conditions can reduce apoptotic cell rate in the cortex and hypothalamus to control level and significantly among them in the frontal cortex. In the thalamus, a significant reduction by DEX under oxygen exposure was demonstrated but did not reach the controls. DEX under normoxic conditions showed a significant increase of TUNEL positive cells in the cortices with DEX10 and in the thalamus with DEX all over (Figure 1(b)). The results of the dentate gyrus show a similar antiapoptotic effect of DEX (data not shown). A statistical evaluation of the dentate gyrus was not possible due to low cell counts.

### 3.2. Treatment with Dexmedetomidine Modifies Hyperoxia-Affected Levels of Reduced (GSH) and Oxidized Glutathione (GSSG) in the Developing Brain

As we showed previously, neonatal oxygen toxicity causes an imbalance of the glutathione-redox-system correlating with an increase of neurodegeneration in the immature brain [12–15].

**Figure 2:**
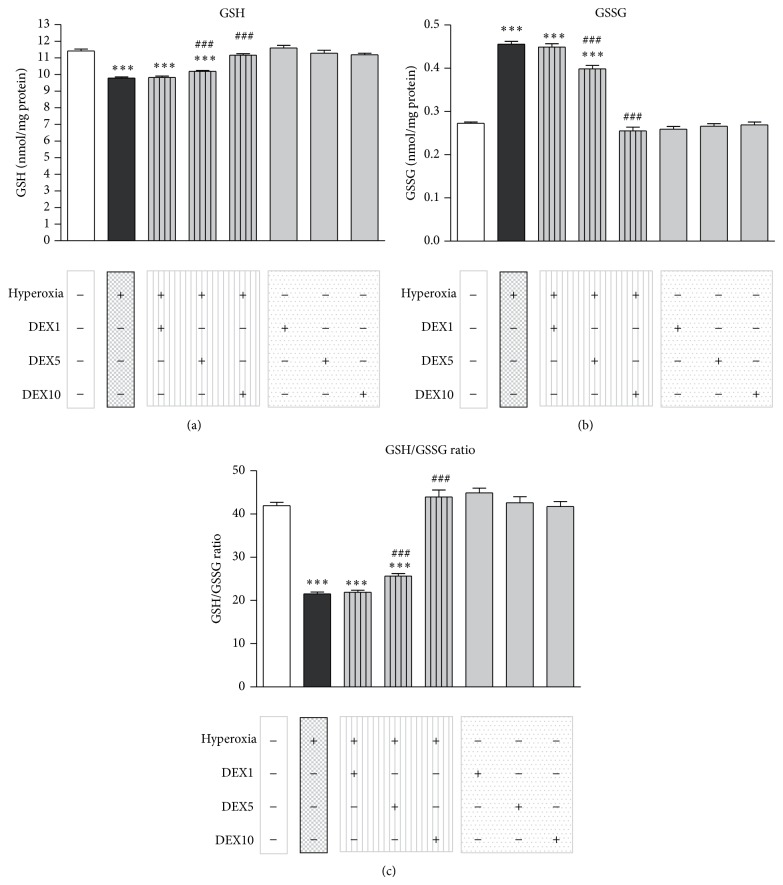
Effect of dexmedetomidine on hyperoxia-modified GSH and GSSG levels in the developing brain. (a) Reduced GSH levels were evident in total rat brain extracts 24 h after the initiation of hyperoxia (black bar) when compared to normoxic animals (white bar). These levels were increased through dexmedetomidine (DEX) pretreatment in a concentration dependent manner (hatched grey bars: 1, 5, and 10 *μ*g/kg). (b) Increased levels of oxidized GSSG were obvious in total brain extracts at 24 h of hyperoxia (black bar) when compared with normoxic control animals (white bar). These levels were decreased through pretreatment with DEX (hatched grey bars: 1, 5, and 10 *μ*g/kg). (c) Reduced GSH/GSSG ratio levels were evident in rat brain extracts at 24 h of hyperoxia (black bar) when compared to normoxic control animals (white bar). These levels were upregulated through DEX pretreatment (hatched grey bars: 1, 5, and 10 *μ*g/kg). Application of dexmedetomidine under room air (grey bars) showed no effect on GSH or GSSG levels. Bars represent mean + SEM; *n* = 6 per group; ^***^ *P* < 0.001 versus normoxia/control; ^###^ *P* < 0.001 versus hyperoxia.

Analysis of GSH and GSSG levels by reaction with the classical thiol reagent DTNB and spectrophotometrical measurement at 412 nm was performed in samples from total brain extracts of 7-day-old rats (*n* = 6 per group) exposed to (i) normoxia and saline injections (CON), (ii) hyperoxia and saline injections (HY), (iii) hyperoxia and DEX (HYDEX; 1, 5, or 10 *μ*g/kg), and (iv) normoxia and DEX (DEX; 1, 5, or 10 *μ*g/kg). 24 h of hyperoxia triggered the decrease of GSH levels in brain extracts (Figure 2(a): CON: 11.42 + 0.11 nmol/mg protein and HY: 9.79 + 0.06 nmol/mg protein). When DEX was administered together with hyperoxia, it significantly increased the levels of GSH at a concentration of 5 and 10 *μ*g/kg DEX (HYDEX5: 10.19 + 0.06 nmol/mg protein and HYDEX10: 11.16 + 0.09 nmol/mg protein).

Figure 2(b) shows the increased level of GSSG in the developing brain after 24 h of hyperoxia (CON: 0.273 + 0.003 nmol/mg protein and HY: 0.046 + 0.007 nmol/mg protein). DEX coapplication reduced the hyperoxia-induced increase of GSSG levels significantly at 5 and 10 *μ*g/kg DEX (HYDEX5: 0.398 + 0.008 nmol/mg protein and HYDEX10: 0.255 + 0.008 nmol/mg protein).

In total, hyperoxia triggered the decrease of the GSH/GSSG ratio in the developing rat brain (Figure 2(c): CON: 41.94 + 0.78 and HY: 21.52 + 0.40). When DEX was administered at the start of hyperoxia, the levels of GSH/GSSG ratio significantly increased at 5 and 10 *μ*g/kg of DEX (HYDEX5: 25.63 + 0.60 and HYDEX10: 43.95 + 1.62).

### 3.3. Induction of Lipid Peroxidation by Hyperoxia in the Neonatal Brain Is Attenuated by Dexmedetomidine Treatment

**Figure 3:**
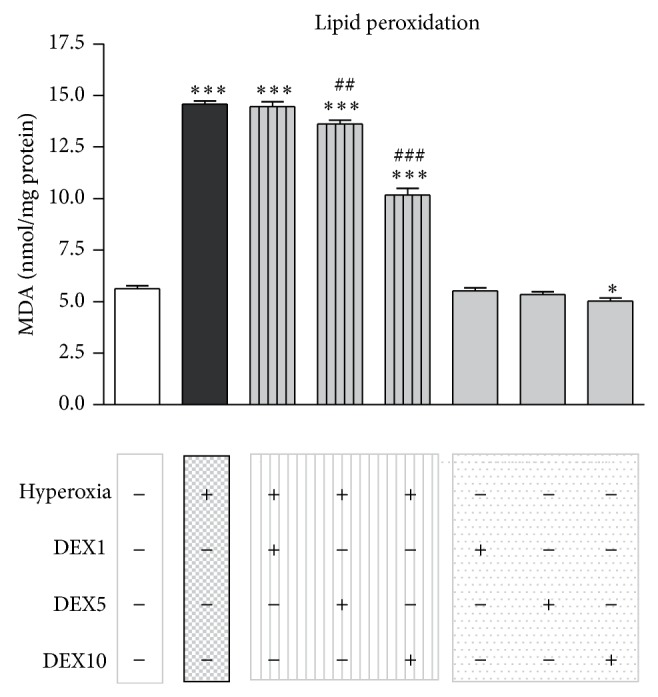
Alteration of lipid peroxidation by hyperoxia in the immature brain. Hyperoxia lead to a significant increase of MDA levels after 24 h of oxygen exposure (black bar), whereas a single DEX application of 5 or 10 *μ*g/kg (hatched grey bars) before hyperoxia exposure reduced these levels significantly. Bars represent mean + SEM, *n* = 6 per group, ^*^ *P* < 0.05 and ^***^ *P* < 0.001 versus normoxia/control; ^##^ *P* < 0.01 and ^###^ *P* < 0.001 versus hyperoxia.

To demonstrate that the changes obtained in the level of GSH and GSSG may be connected with an altered lipid peroxidation, we examined immature rat brains after treatment of hyperoxia, normoxia, dexmedetomidine, and/or normal saline treatment for the concentration of malondialdehyde (MDA). Increased MDA levels, as a sign of lipid breakdown, were evident in rat brains at 24 h of hyperoxia when compared to normoxic animals, whereas a single DEX administration before beginning hyperoxia exposure reduced these levels significantly at 5 and 10 *μ*g/kg DEX (Figure 3: CON: 5.63 + 0.14 nmol/mg protein and HY: 14.57 + 0.16 nmol/mg protein; HYDEX5: 13.62 + 0.19 nmol/mg protein and HYDEX10: 10.18 + 0.31 nmol/mg protein).

### 3.4. Dexmedetomidine Treatment Reduces IL-1*β* Expression under Hyperoxic Conditions in the Infant Brain

As previously shown by our group hyperoxic conditions lead to an increase in IL-1*β* expression in the immature brain [13, 15, 16].

**Figure 4:**
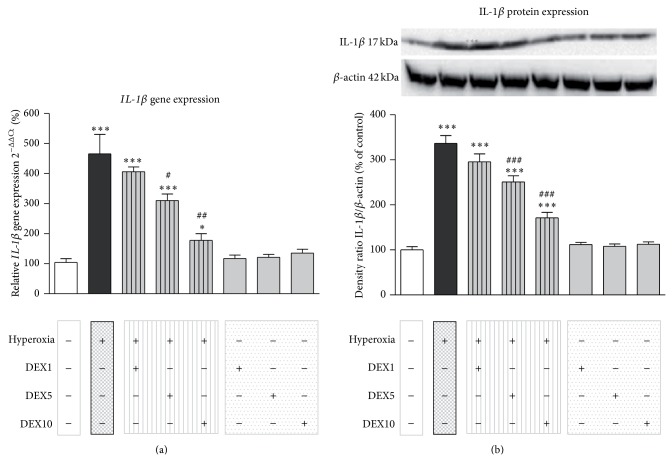
(a) Quantitative analysis of mRNA expression by real-time PCR showed a marked increase of* IL*-*1β* mRNA expression in the brain of P6 rat pups that were kept for 24 h under hyperoxia (black bar), whereas dexmedetomidine treatment restores* IL*-*1β* upon control level (hatched grey bars) depending on the dexmedetomidine concentration. Application of dexmedetomidine under room air (grey bars) showed no significant regulation on* IL*-*1β* mRNA expression. (b) The analysis of IL-1*β* protein expression by western blot showed a similar expression pattern. The protein expression of IL-1*β* is significantly increased after 24 h of hyperoxia and a single application of 5 or 10 *μ*g/kg dexmedetomidine could restore the IL-1*β* protein expression almost up to control level. The densitometric data represent the ratio of the pixel intensity of the IL-1*β* band to the corresponding *β*-actin band. Blots are representative of a series of three blots. Data are normalized to levels of rat pups exposed to normoxia (CON = 100%, white bars). Bars represent mean + SEM; *n* = 6 per group; ^***^ *P* < 0.001 versus normoxia/control; ^#^ *P* < 0.05, ^##^ *P* < 0.01, and ^###^ *P* < 0.001 versus hyperoxia.

Hyperoxia triggered an increase of* IL*-*1β* mRNA in brain homogenates of neonatal rats as shown by semiquantitative real-time PCR (Figure 4(a): HY: 465.99 + 64.69%). Control animals showed low mRNA levels of* IL*-*1β* (CON: 100.36 + 11.41%). When dexmedetomidine was administered together with hyperoxia, it significantly ameliorated the expression of proinflammatory* IL*-*1β* depending on the DEX concentration (HYDEX5: 310.29 + 21.77% and HYDEX10: 177.66 + 22.33%) on mRNA levels in rat pups. Pretreatment with DEX in animals kept under normoxic conditions had no effect on* IL*-*1β* gene expression (DEX1: 117.18 + 11.58%, DEX5: 121.41 + 9.14%, and DEX10: 135.31 + 12.85%).

Western blotting demonstrated that protein expression of IL-1*β* is significantly increased following oxygen treatment for 24 h (Figure 4(b): HY: 336.66 + 17.25%) compared to control animals (CON: 100.00 + 7.31%). A significant effect on IL-1*β* protein levels was seen upon a single dexmedetomidine application of 5 or 10 *μ*g/kg DEX in hyperoxia-exposed animals (HYDEX5: 250.96 + 13.55% and HYDEX10: 171.05 + 12.27%). Animals under normoxic conditions and DEX pretreatment showed low protein levels of IL-1*β* (DEX1: 111.95 + 4.43%, DEX5: 107.86 + 5.15%, and DEX10: 112.44 + 5.43%).

## 4. Discussion

In the present study, we demonstrate that the *α*2-adrenoceptor agonist dexmedetomidine leads to a decrease of neurodegeneration and affects the level of oxidative stress parameters and the proinflammatory cytokine IL-1*β* in a model of neonatal hyperoxia-induced brain injury.

As shown previously oxygen toxicity leads to an increased neurodegeneration in the immature brain [12, 16, 17, 38]. In line with these studies here we indicate that an exposure of six-day-old rats to a high oxygen concentration (FiO_2_ 80%) over 24 h resulted in an increase of neurodegeneration to the neonatal brain (Figures 1(a) and 1(b)). Moreover, we point out a significant decrease of cell death in the developing rat brain when infant rats were treated intraperitoneally with 1, 5, or 10 *μ*g/kg dexmedetomidine before exposure to high oxygen conditions. These dosages are at/under the lower levels of dexmedetomidine concentrations used in common adult rat models of neuronal damage [39, 40] and the present data are not intended to demonstrate the evidence of an effective dose. However, our findings conform with different other animal studies which examined the neuroprotective capacity of dexmedetomidine [22–27]. Hyperoxia-exposed rats revealed an up to 5-fold increase in cell degeneration within the cortex and deep gray matter (Figure 1(b)), which has been demonstrated in previous studies [17, 41]. Interestingly, dexmedetomidine treatment showed a profound decrease of degenerating cells in all analysed brain regions of rats exposed to hyperoxia (Figure 1(b)). Dexmedetomidine alone induced no oxidative stress, but at a relatively low concentration of dexmedetomidine under normoxic conditions, a significant increase in apoptosis in the cortex and the thalamus has been shown. This result is controversial to other inventions [31, 32, 42] and to the description of only protective and no toxic effects [24, 25, 43].* In vitro* it was shown by Kuhmonen et al. [22] that dexmedetomidine is more effective at lower doses and protects against delayed cell death. In an ischemia-reperfusion model [39] antiapoptotic proteins were upregulated and proapoptotic proteins are suppressed by dexmedetomidine treatment. In the study by Li et al. there are no changes in the expression of proapoptotic mediators in an isoflurane-induced model of neuroapoptosis [40]. A proven high blood pressure after the administration of high-dose dexmedetomidine correlates with cerebral hypoperfusion may be due to alpha-2-induced cerebral vasoconstriction [44], so that this could contribute to apoptotic cell death triggered by other cellular mechanisms. However, in our study dexmedetomidine also led to negative effects in control animals notably in the thalamus, and potential side effects should be further considered in studies on protection of the immature brain using dexmedetomidine.

The potency of dexmedetomidine in this model of oxygen toxicity indicates that the *α*2-adrenoceptor agonist has antioxidant activities. Of note, our hyperoxia model is predominantly based on free radical generation, as we previously found an induction of different oxidative stress parameters [12–15]. Therefore, we evaluated the effects on biochemical markers of oxidative stress in the developing brain. The concentrations of GSH and GSSG, the GSH/GSSG ratio, and the MDA level were used as indicators of oxidative stress and lipid breakdown. Hyperoxia-exposed P6 rats showed significant decreased GSH and increased GSSG levels at 24 h of hyperoxia. In addition, a decrease in GSH/GSSG ratio and an increase in MDA level in the developing brain indicate neuronal damage due to oxidative stress, but there was a modulation of the levels of all parameters up to normoxia levels in hyperoxia-exposed neonatal rats that received dexmedetomidine (Figures 2 and 3). These observations suggest that dexmedetomidine seemingly has an antioxidant activity against hyperoxia-induced oxidative stress in the developing brain. In line with our findings there are several studies that suggest an increased antioxidative capacity of dexmedetomidine in the brain [24] and further organs (reviewed by Tse et al. [45]).

This study indicates that dexmedetomidine significantly decreases hyperoxia-induced IL-1*β* upregulation in the developing rat brain. These results illustrate the protective effect of dexmedetomidine on neuroinflammation as shown before in clinical and experimental studies [46, 47]. Dexmedetomidine itself did not affect IL-1*β* levels, indicating that the *α*2-adrenoceptor agonist only acts to normalize the induced proinflammatory cytokine (Figure 4). Here, dexmedetomidine appears to inhibit the production of inflammatory mediators from multiple cell types, for example, astrocytes and microglia [48, 49].

There are several limitations of this study pointing to areas of future investigations. Dexmedetomidine was given only once to the animals, while preterm infants receive an initial loading dose and subsequent infusions [50] and the high doses of dexmedetomidine studied here exceed those doses recommended for clinical use; short term hyperoxia was examined and preterm infants are often exposed to a longer period of supraphysiological oxygen concentrations [51], and we have not determined the plasma levels of dexmedetomidine. Further details of dexmedetomidine-induced side effects remain to be elucidated.

To our knowledge this is the first report demonstrating that dexmedetomidine mediates a decrease of neurodegeneration and oxidative stress and affects the expression of the proinflammatory cytokine IL-1*β* in the hyperoxia-exposed neonatal brain. Since oxidative stress and inflammation are involved in dysfunction of the immature brain under high oxygen conditions, *α*2-adrenoceptor activation seems to be a potential neuroprotective treatment.

## 5. Conclusion

The essential core of this work is the finding that dexmedetomidine prevents oxidative stress, inflammation, and cell death in the neonatal brain under hyperoxic conditions. Exposure to high oxygen in the developing brain of six-day-old rat pups leads to increased oxidative stress-induced and inflammatory DNA damage, as demonstrated by an increased apoptotic rate, a reduction of the GSH/GSSG ratio, increased lipid peroxidation, and increased IL-1*β* levels. Remarkably, a single dose of dexmedetomidine has weakened or abolished all these detrimental effects under hyperoxic conditions. However, in this study dexmedetomidine also shows negative effects in the control animals, and these potential side effects should be taken into consideration in further studies on the protection of the immature brain with dexmedetomidine.

Based on these data the hyperoxic model indicates dexmedetomidine as a sedative in pediatric anesthesia and a possible promising agent for neuroprotective strategies in preterm infants.
